# Influence of Pre-Sintered Zirconia Surface Conditioning on Shear Bond Strength to Resin Cement

**DOI:** 10.3390/ma9070518

**Published:** 2016-06-25

**Authors:** Tomofumi Sawada, Sebastian Spintzyk, Christine Schille, Judit Zöldföldi, Angelos Paterakis, Ernst Schweizer, Ingrid Stephan, Frank Rupp, Jürgen Geis-Gerstorfer

**Affiliations:** 1 Section Medical Materials Science & Technology, University Hospital Tübingen, Osiander Strasse 2-8, Tübingen 72076, Germany; Sebastian.Spintzyk@med.uni-tuebingen.de (S.S.); christine.schille@med.uni-tuebingen.de (C.S.); ernst.schweizer@med.uni-tuebingen.de (E.S.); Ingrid.Stephan@med.uni-tuebingen.de (I.S.); frank.rupp@med.uni-tuebingen.de (F.R.); geis-gerstorfer@mwt-tuebingen.de (J.G.-G.); 2 Department of Prosthodontics, Center of Dentistry, Oral Medicine, and Maxillofacial Surgery, University Hospital Tübingen, Osianderstrasse 2-8, Tübingen 72076, Germany; Angelos.Paterakis@med.uni-tuebingen.de; 3 Institute for Materials Science, University of Stuttgart, Pfaffenwaldring 2b, Stuttgart 70569, Germany; judit.zoeldfoeldi@mpa.uni-stuttgart.de

**Keywords:** zirconia, yttria-stabilized tetragonal zirconia polycrystal (Y-TZP), adhesion promoter, shear bond strength, surface modification, resin cement

## Abstract

This study analyzed the shear bond strength (SBS) of resin composite on zirconia surface to which a specific conditioner was applied before sintering. After sintering of either conditioner-coated or uncoated specimens, both groups were divided into three subgroups by their respective surface modifications (*n* = 10 per group): no further treatment; etched with hydrofluoric acid; and sandblasted with 50 µm Al_2_O_3_ particles. Surfaces were characterized by measuring different surface roughness parameters (e.g., *R_a_* and *R_max_*) and water contact angles. Half of the specimens underwent thermocycling (10,000 cycles, 5–55 °C) after self-adhesive resin cement build-up. The SBSs were measured using a universal testing machine, and the failure modes were analyzed by microscopy. Data were analyzed by nonparametric and parametric tests followed by *post-hoc* comparisons (α = 0.05). Conditioner-coated specimens increased both surface roughness and hydrophilicity (*p* < 0.01). In the non-thermocycled condition, sandblasted surfaces showed higher SBSs than other modifications, irrespective of conditioner application (*p* < 0.05). Adhesive fractures were commonly observed in the specimens. Thermocycling favored debonding and decreased SBSs. However, conditioner-coated specimens upon sandblasting showed the highest SBS (*p* < 0.05) and mixed fractures were partially observed. The combination of conditioner application before sintering and sandblasting after sintering showed the highest shear bond strength and indicated improvements concerning the failure mode.

## 1. Introduction

Zirconia-based all-ceramic restorations have been used successfully in dental clinics along with dental computer-aided design/computer-aided manufacturing (CAD/CAM) systems [1]. Zirconia is one of the most promising restorative materials because it shows very favorable mechanical properties and reasonable esthetics [2]. The clinical performance of zirconia-based fixed partial dentures (FPDs) showed a similar survival rate compared with that of metal-ceramic FPDs in the short- and medium-term [3,4]. Unfortunately, biological and technical complications have been generally confirmed, not only for all-ceramic restorations, but also for metal-ceramic restorations. The most common complications in bilayered porcelain/zirconia crowns have been identified as loss of retention, endodontic treatment, fractures of veneering ceramics, and bleeding on probing [5]. Moreover, chipping of veneering ceramics on zirconia-based FPDs was higher compared with that of metal-ceramic FPDs [6]. Likewise, in in vitro reports, the bond strength between metal and veneering ceramics was higher compared with that between zirconia and veneering ceramics [7].

For achieving long-term clinical success, various approaches have attempted the improvement of the bonding between zirconia and either veneering ceramics or cement materials [8,9,10,11,12,13]. Modification of the zirconia framework and sandblasting (airborne abrasion) of zirconia surfaces have been recommended to improve the fracture resistance of veneering ceramics [8,9]. On the other hand, for bonding of zirconia to cement materials, an in vitro systematic review summarized seven surface treatment methods with 23 different procedures [10]. Generally, a tribochemical method such as sandblasting with alumina (Al_2_O_3_) particles has been used to adapt the zirconia surface [11,12]. Furthermore, silanization by silane coupling agents or universal adhesive primers after silicatization has improved zirconia bonding to cement materials [11]. The combination of sandblasting followed by a universal primer contributed to zirconia bonding with resin cements that contain phosphate ester monomer 10-methacryloyloxydecyl dihydrogen phosphate (MDP) [12]. However, shear bond strength (SBS) was influenced by Al_2_O_3_ particle sizes and universal primer products [13]. A recent systematic review summarized the correlation between in vitro studies and clinical studies [14]. This report indicated well-established clinical evidence that sandblasting at a moderate pressure and using primers containing phosphate monomer and/or luting resins provide long-term durable bonding to zirconia. In addition, alternative approaches i.e., laser, acid, and low-fusing glaze porcelain coating treatments have been introduced [15,16,17]. However, the clinical evidence of these alternative methods is yet unclear. Kern [14] suggested that the aforementioned alternative bonding systems might be reconsidered because these methods were more time consuming or require rather complicated and technique sensitive procedures.

A simple alternative approach was developed utilizing a novel conditioner (mixture of silicate ceramic and quartz) to pre-sintered zirconia for promoting the adhesion between oxide ceramics and veneer ceramics [18]. Our previous study has already shown a decrease of adhesive fractures between zirconia coated with this novel conditioner and veneering ceramics [19]. This procedure can save working time because an experimental conditioner is just applied on the zirconia surface before sintering. If this conditioning method would also improve the bonding between zirconia and cement material at the same time, this might reduce both major clinically relevant technical complications i.e., fracture of veneering ceramics and loss of prostheses retention. Thus, the evaluation of the bonding between zirconia coated with this novel conditioner and cement material is needed.

Therefore, the aim of this study was to analyze bonding via SBS testing between conditioner-coated zirconia surfaces modified with three different treatments and resin cement. The null hypotheses were that the conditioner-coated zirconia followed by either sandblasting or etching would neither improve the shear bond strength nor the failure mode, irrespective of thermocycling.

## 2. Materials and Methods

### 2.1. Material Characterization of an Experimental Conditioner

An experimental conditioner (Luxor Zirkonoxyd-Primer, Xplus 3 GmbH, Echzell, Germany) was used. The dried powder of this experimental conditioner was pulverized again and performed to identify the crystalline phases by X-ray diffraction (XRD) analysis using a diffractometer (D8, Bruker AXS GmbH, Karlsruhe, Germany) with Cu-K α-radiation (λ = 1.54 Å) and variable divergence slits at convention reflection geometry in 5°–70° 2θ angle range.

In addition, X-ray fluorescence (XRF) analysis was performed to determine the chemical composition using a wavelength dispersive X-ray spectrometer (S4 PIONEER, Bruker AXS GmbH, Karlsruhe, Germany) at 4 kW.

### 2.2. Preparation of Zirconia Specimens

**Figure 1:**
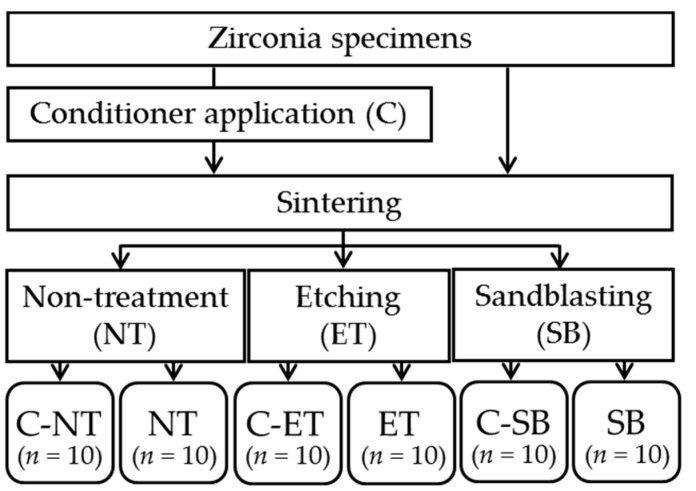
Study design. Abbreviations of each experimental group are shown in Table 2, respectively.

Table 1 shows the materials used in this study. Rectangular yttria-stabilized tetragonal zirconia polycrystal (Y-TZP) specimens (25 × 12 × 2.5 mm^3^) were fabricated from pre-sintered zirconia blanks (Nacera Pearl 1, Doceram GmbH, Dortmund, Germany) using a CAD/CAM system. The cut specimens (*n* = 60) were polished with #320 and #1200 silicon carbide paper (CarbiMet, Buehler GmbH, Düsseldorf, Germany). An experimental conditioner [18] was applied to half of the specimens before sintering (conditioner-coated specimens (C), *n* = 30). Specifically, a stirred slurry was coated to Y-TZP surfaces twice using a thick brush. The conditioner-coated specimens and the remaining uncoated specimens (*n* = 30) were sintered in a furnace (VITA ZYrcomat, VITA Zahnfabrik GmbH, Bad Säckingen, Germany) according to manufacturer’s instructions. Both types of specimens were divided into three subgroups after sintering, respectively (Figure 1, Table 2); Surfaces were etched with 9.5% HF (Porcelain Etch, Ultradent products GmbH, Köln, Germany) for 90 s (ET), or sandblasted with 50 µm Al_2_O_3_ particles (Spezial-Edelkorund Klasse 30B/50 my, Harnisch + Rieth GmbH, Winterbach, Germany) at 0.2 MPa of air pressure for 20 s (SB), or they remain non-treated as a control group (NT). Sandblasting was performed vertically at a fixed distance of 20 mm between the specimen surface and the nozzle using a special metal holder. After each surface modification, all specimens (20 × 10 × 2.0 mm^3^) were ultrasonically cleaned in 70% ethanol for 10 min, followed by distilled water cleaning and air-drying.

### 2.3. Surface Characterization

The surfaces were characterized using three different analytical methods. Surface roughness measurements were conducted with a contact profilometer (Perthometer SP6, Mahr GmbH, Göttingen, Germany). The measurement area was set to 3 × 3 mm^2^ with 121 profiles. The profile roughness parameters were measured *R_a_* (mean roughness; the arithmetic average of the absolute values of the roughness profile ordinates), *R_max_* (maximum roughness depth), *R_sk_* (skewness) [20], and the *R_pm_*/*R_z_* ratio (profile shape; *R_pm_* is the average of the leveling depths of five consecutive lengths, and *R_z_* is the mean roughness depth) [21].

For half of the specimens in each experimental group (*n* = 5), water contact angles were measured using a drop shape analysis system (DSA10-MK2, KRÜSS GmbH, Hamburg, Germany) at room temperature (25 °C). A drop of water (2 µL) was applied to the zirconia surface with a syringe and the drop shape was observed by a video camera for 30 s. Two drops were analyzed on each specimen and the mean contact angle was calculated from 20 s old sessile drops.

Subsequently, the surfaces were sputter-coated with carbon (Kohlegarn, Baltic Präparation e.K, Niesgrau, Germany). Qualitative analyses were performed to evaluate the surface morphology by scanning electron microscopy (SEM; LEO 1430, Carl Zeiss AG, Oberkochen, Germany) along with energy dispersive X-ray spectroscopy (EDX) for an elemental analysis at 10 kV.

### 2.4. Bonding of Resin Cement

Before bonding to cement, a universal primer (Monobond Plus, Ivoclar Vivadent GmbH, Ellwangen, Germany) was applied to the surface of each specimen with a microbrush, allowed to react for 60 s. Then, any remaining excess was dispersed with a strong steam of air. A self-adhesive resin cement (Panavia SA Cement Plus Automix, Kuraray Europa GmbH, Hattersheim am Main, Germany) was built up on zirconia surfaces using a special brass jig (20 × 10 × 2.0 mm^3^) with a hole (5 mm in diameter) (*n* = 10). Polymerization was used with a dual curing method (chemical and light curing) according to manufacturer’s instructions. Light curing was performed with a light emitting diode (LED) curing device (1200 mW/cm^2^; Bluephase 20i, Ivoclar Vivadent GmbH, Ellwangen, Germany) for 40 s. After polymerization, the mold was lifted and removed carefully. All specimens were stored in an incubator at 37 °C for 24 h. Prior to SBS testing, half of the specimens from each experimental group (*n* = 5) [22] underwent 10,000 thermal cycles, which was estimated to represent 7 days water saturation between 5 °C and 55 °C (70 s per cycle; dwelling time: 30 s, transfer time: 5 s) (Table 2).

### 2.5. Shear Bond Strength Testing

The SBSs of all specimens with (+) or without thermocycling were measured on a universal testing machine (Z010, Zwick GmbH, Ulm, Germany) at a crosshead speed of 1.0 mm/min until debonding occurred. The bond strength was calculated in MPa using the maximum fracture load (N) divided by the area (mm^2^) of the resin [23]. Failure modes were analyzed by using stereomicroscopy (Wild Heerbrugg AG, Heerbrugg, Switzerland) at 10× magnification or by SEM. Subsequently, failure modes were defined and classified into three different fracture patterns: adhesive, less than 20% of the cement remained on the zirconia surface; mixed, more than 20% but less than 80% of the cement remained on the zirconia surface (a combination of adhesive and cohesive fractures); and cohesive, more than 80% of the cement remained on the zirconia surface [24].

### 2.6. Statistical Analysis

All data were analyzed for normal distribution by Shapiro–Wilk test and for equality of the variances by Levene test. Descriptive statistics were applied using medians for surface roughness parameters and SBSs, and means and standard deviations for water contact angles. The surface roughness and SBS results were analyzed by non-parametric Kruskal–Wallis and Mann–Whitney U tests. The contact angle results were analyzed by a two-way analysis of variance (ANOVA) followed by Tukey’s test for post hoc comparisons. The statistical analyses were performed by the software package (Excel Statistics 2010, Social Survey Research Information Co., Ltd., Tokyo, Japan) at a level of significance of α = 0.05.

## 3. Results

### 3.1. Structure and Composition of the Conditioner

**Figure 2:**
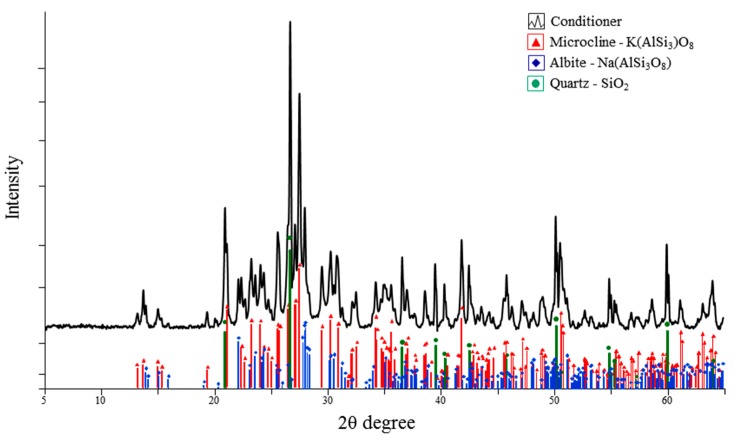
X-ray diffraction (XRD) analysis of the dried powder.

The XRD analysis of the dried powder showed that this conditioner mainly consisted of quartz, K-feldspar (microcline) and Na-feldspar (albite) (Figure 2). The quantitative analysis by XRF confirmed these crystalline components (Table 3).

### 3.2. Surface Characterization of Zirconia Specimen

The surface roughness results are shown in Table 4. The medians of *R_a_* and *R_max_* values in the conditioner-coated specimens were significantly higher than those in the conditioner-uncoated specimens (*p* = 0.002), except in the ET group. There were no significant differences among the subsequent surface modifications in the conditioner-coated specimens, whereas the NT group showed the lowest values that were significantly lower than the SB and ET groups in the conditioner-uncoated specimens (*p* = 0.002 and *p* = 0.018). The *R_sk_* and *R_pm_*/*R_z_* values in the conditioner-coated specimens were significantly higher than those in the conditioner-uncoated specimens (*p* < 0.05). In the conditioner-coated specimens, the *R_sk_* and *R_pm_*/*R_z_* values in the C-NT and C-ET groups were significantly higher than those in the C-SB group (*p* = 0.002). There was no significant difference between C-NT and C-ET groups. In the conditioner-uncoated specimens, the *R_sk_* and *R_pm_*/*R_z_* values in the NT group was significantly higher than those in the SB group (*p* = 0.002), and no significant difference was observed compared with those in the ET group.

**Figure 3:**
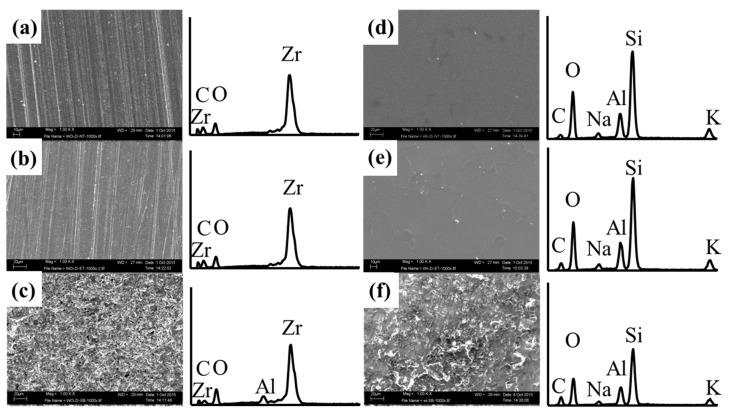
Scanning electron microscopy (SEM) micrographs (**left**; 1000× magnification) and energy dispersive X-ray spectroscopy (EDX) analysis (**right**) from each experimental group: (**a**) NT; (**b**) ET; (**c**) SB; (**d**) C-NT; (**e**) C-ET; and (**f**) C-SB. Abbreviations of each experimental group are shown in Table 2, respectively

**Figure 4:**
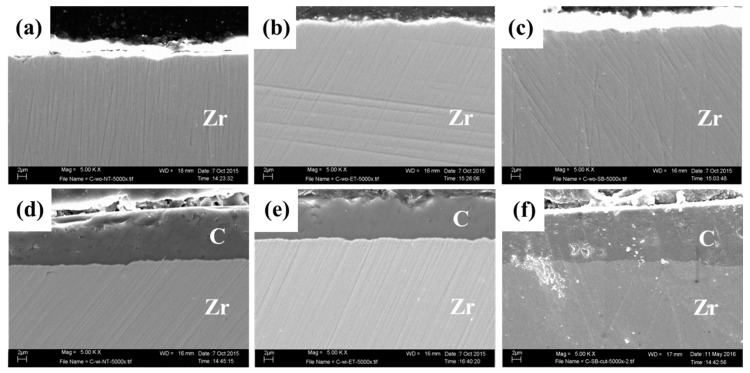
Scanning electron microscopy (SEM) micrograph (5000× magnification) from each experimental group with cross-sectional view. (**a**) NT; (**b**) ET; (**c**) SB; (**d**) C-NT; (**e**) C-ET; and (**f**) C-SB. Abbreviations of each experimental group are shown in Table 2, respectively. Zr, zirconia; and C, conditioner layer.

The SEM-EDX analyses are shown in Figure 3 and Figure 4. In the conditioner-uncoated specimens, polishing traces were observed on the surfaces of the NT and ET groups, and the chemical elements consisted of zirconia, oxygen, and carbon in the EDX spectra (Figure 3a,b). In contrast, polishing traces were not observed after sandblasting, and aluminum was further detected in the surface of the SB group (Figure 3c). In the cross-sectional images of the conditioner-coated specimens, all surface modifications were similar (Figure 4a–c).

The zirconia surfaces on the C-NT and C-ET groups showed a coating layer that was constant in thickness, and polishing traces were not detected in the conditioner-coated specimens (Figure 3d,e). The C-SB group was further covered with Al_2_O_3_ particles and the conditioner layer was not observed (Figure 3f); however, a cross-sectional image showed the presence of a conditioner coating layer under the sandblasted surface (Figure 4f). The chemical elements (O, C, Al, Si, Na, K) in the conditioner-coated specimens were similar as shown in the EDX spectra (Figure 3d–f, right).

### 3.3. Contact Angle Measurement

The water contact angles measured on each surface type are shown in Table 5. The contact angles in the conditioner-coated specimens were significantly lower than those in the conditioner-uncoated specimens (*p* < 0.001). There were no significant differences among the subsequent surface modifications (*p* = 0.473).

### 3.4. Shear Bond Strength (SBS)

**Figure 5:**
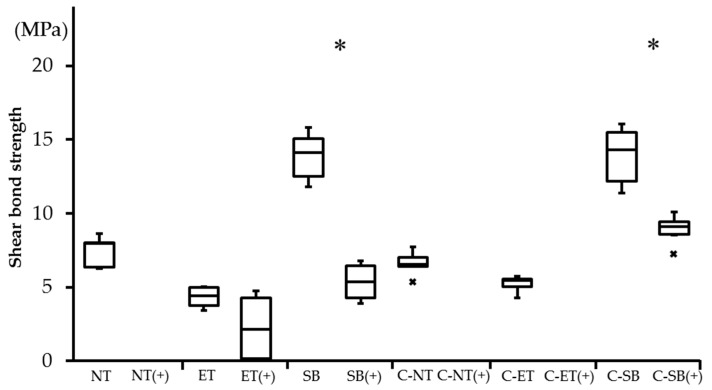
Box-plots of shear bond strengths in the different experimental groups. Asterisks indicate significant differences between the non-thermocycled and the thermocycled groups (*p* < 0.05). Abbreviations of each experimental group are shown in Table 2, respectively. (+) indicates thermocycled samples.

The SBS values are shown in Table 6 and Figure 5. Without thermocycling, there were no significant differences between the conditioner-coated and the uncoated groups. The SBS values of the C-SB and SB groups were significantly higher than those of other groups (*p* = 0.0038), whereas the C-ET and ET groups showed the lowest SBS without thermocycling.

Pre-failure was observed in all experimental groups during thermocycling, but differed in number. In particular, all specimens were observed cement debonding in the NT (+), C-NT (+), and C-ET (+) groups. These specimens were excluded from subsequent SBS testing and defined as 0 MPa [13]. With thermocycling, the SBS values of the C-SB (+) group were significantly higher than those of the ET (+) and SB (+) groups (*p* = 0.039).

For the influence of thermocycling, the SBS values showed significant decreases in the SB (+) and C-SB (+) groups, respectively (*p* = 0.034, *p* = 0.014) (Figure 5).

### 3.5. Analysis of Failure Modes

**Figure 6:**
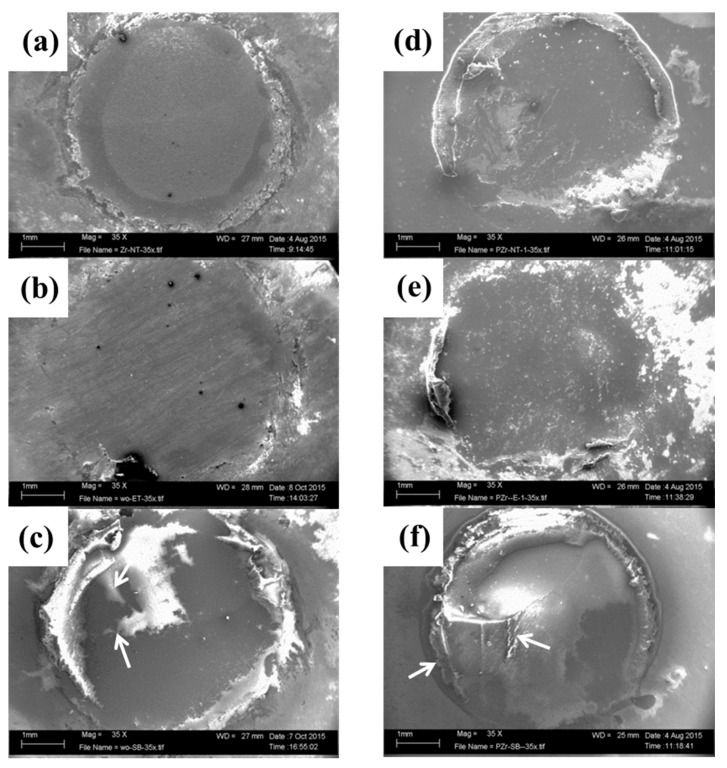
SEM micrograph (35× magnification) from each experimental group without thermocycling. (**a**) NT; (**b**) ET; (**c**) SB; (**d**) C-NT; (**e**) C-ET; and (**f**) C-SB. Abbreviations of each experimental group are shown in Table 2, respectively. (+) indicates thermocycled samples. White arrows indicate cement residues.

The classifications of failure modes are shown in Table 7 and Figure 6. Adhesive fractures were observed in all experimental groups without thermocycling; however, the C-SB group additionally showed 40% mixed fractures. Although a small amount of cement remained on the surface of the SB and C-SB groups (Figure 6c,f), cement was not remained on the entirety surfaces of other groups. After thermocycling, complete adhesive fractures were also observed in the ET (+) and SB (+) groups; however, the C-SB (+) group differed in percentages (adhesive, 75% and mixed, 25%).

## 4. Discussion

The focus of this study was to evaluate the effect of a specific conditioner application to pre-sintered zirconia surfaces on bonding to resin cement. The terms conditioner and primer are not clearly defined in the literature. In our study, the term conditioner is strictly used for the pre-sintered stage. The term primer is used in the field of cement bonding only. Without thermocycling, SBS values in the sandblasted specimens (the C-SB and SB groups) were higher than those in other groups. Adhesive fractures were commonly observed in the specimens, except in the C-SB group. After thermocycling, all groups showed significantly decreased SBS values; however, the C-SB (+) group showed the highest SBS value. In addition, adhesive fractures occurred in all groups; however, the C-SB (+) group partially showed mixed fractures. Thus, the first and second null hypotheses were rejected.

Conditioner application affected the surface roughness parameters and changed the morphology. For the surface roughness parameter *R_a_* and *R_max_*, the conditioner-coated specimens showed significantly higher values than the conditioner-free specimens. In particular, the C-NT group showed a four-fold rougher surface than the NT group. Other surface roughness parameters (*R_sk_* and *R_pm_*/*R_z_*) were also used to clarify the surface morphology. The *R_sk_* and *R_pm_*/*R_z_* values in the C-NT group were significantly higher than those in the NT group (Table 4). From these results, the surface of the C-NT group was characterized as a sharp, rough surface. Our *R_a_* results of the C-NT group are in agreement with those of a previous study [25]. An experimental glass slurry coated onto zirconia surfaces before sintering caused a significant increase in *R_a_* because the slurry contents adhered to the surfaces after sintering. The experimental conditioner, studied here, contained silicate ceramics and quartz as shown by XRD and XRF (Figure 2, Table 3), and was adhered onto the surfaces of the C-NT group as confirmed by SEM-EDX (Figure 3d).

Moreover, conditioner-coated specimens affected the water contact angles which were significantly decreased compared with conditioner-free specimens. This finding may be supported by a previous study showing that the contact angle of a Si-based thin film coated zirconia surface was significantly lower than those of non-coated and silica-modified Al_2_O_3_-sandblasted surfaces [26]. Their film coating by magnetron sputtering technique could obtain a strong chemical bonding to the zirconia surface as a homogeneous layer without a change in surface roughness. In contrast, our application method induced a rough surface and decreased the contact angle in the C-NT group. This difference was due to the application system. In our study, slurry conditioner components were not uniformly distributed on the zirconia surface by manual coating. This resulted in weaker chemical bonding compared with a film coating method, but led to the micromechanical retention by a rough surface.

Morphological changes occurred on both conditioner-coated and uncoated specimens by the respective subsequent surface modifications. Sandblasting has been recognized as a standard modification for zirconia in both in vitro and clinical systematic reviews [10,14]. In general, sandblasting which caused an increase of surface roughness and modified the wettability, improves the micromechanical retention [13]. Surface roughness in the SB group increased the *R_a_* and *R_max_* values compared with those in the NT group. These findings were confirmed by the SEM-EDX results; Al, which is a component of the sandblasting particles, was detected on the surfaces of the SB group, whereas it was not detected on the surfaces of the NT group (Figure 3a,c). This behavior was also reflected by other surface roughness parameters, showing a negative skew (*R_sk_*: <0), and changed the rounded profile (*R_pm_*/*R_z_*: <0) in the SB group. These tendencies of surface changes also occurred in the C-SB group. The C-SB group showed significantly lower *R_sk_* and *R_pm_*/*R_z_* values compared with the C-NT group although there was no significant difference in *R_a_* and *R_max_* values. From these findings, the conditioner layer was diminished, but still could be observed in the surface of the C-SB group after our moderate sandblasting procedure (Figure 4f).

HF etching is a conventional method to change the microstructure and obtain the appropriate microstructure for bonding in feldspathic ceramics [27]. In contrast, the dense crystal network of zirconia is resistant to chemical treatments [28]. However, recent reports have shown that zirconia can be etched under certain conditions i.e., a high HF concentration (48%), a long application time (0.5–24 h), and high temperature (80 °C) [16,29]. Our results of *R_a_* and *R_max_* in the ET group indicated increased surface roughness compared with those in the NT group. In the C-ET group, etching had only a minor effect on the conditioner layer, as shown by the SEM-EDX analyses and all surface roughness parameters that were similar to those in the C-NT group. Thus, our results show that the conditioner application influenced the surface characteristics more than sandblasting or etching.

The SBS values of the sandblasted specimens exhibited higher strength than those of etched or non-treated specimens in the non-thermocycled condition, irrespective of conditioner application. In particular, the ET and C-ET groups showed the lowest SBS. These results were in agreement with a previous report by Smielak et al. [30]. The surface roughness (*R_a_*) of zirconia changed at the nanometer level after 40% HF etching for 15 min, which might have resulted in less bonding to luting resin cements. Sriamporn et al. [16] reported that the lower bond strength could be explained by the high viscosity of the resin cement, which cannot penetrate into the nano-pores of the etched zirconia surface. Furthermore, a tetragonal-to-monoclinic phase transformation was induced in the etched zirconia surface, which may lead to crack propagation and reduced mechanical properties [16,31]. In our study, a micromechanical bond could not be obtained completely between the nano scaled zirconia surface and resin composite in the ET and C-ET groups.

Conversely, in the SB and C-SB groups, an explanation may be that resin cement can penetrate into the micro-scaled surface of the sandblasted zirconia to obtain micromechanical interlocking in contrast to etching. This positive effect is dependent on the sandblasting conditions, i.e., the blasting material, particle size, and application time. Using 50 µm Al_2_O_3_ particles at a moderate pressure (0.25 MPa) was recommended on clinical evidence [14]. Our sandblasting condition was similar with their report. However, adhesive fractures were observed in the SB group in spite of the presence of the residual resin cement (Figure 6c), whereas mixed fractures were partially observed in the C-SB group. This difference may be caused by conditioner application and by sandblasting because micromechanical interlocking and chemical bonding in the silica-free zirconia surface (the SB group) seemed to be insufficient compared with those in the C-SB group. In general, a strong resin bonding relies on micromechanical interlocking and adhesive chemical bonding to the ceramic surface, requiring surface roughening for mechanical bonding and surface activation for chemical adhesion [30].

Thermocycling or/and long-term water storage were used as conditions to simulate the aging of resin bonding [32]. Water may penetrate the network and cause the composite to expand. This state is reached when the weight of the material stops increasing. It may take several weeks until a material has expanded to its maximum. According to ISO 4049 [33] water sorption must be below 40 µg/mm^3^ after seven days. In many bond strength studies, a preliminary storage in water is carried out 24 h [10]. In this study, time for water absorption during thermocycling was seven days. Even if a full water absorption has not been reached, the aging process performed here gives a first indication of the long-term stability. Different degrees of debonding were observed in all experimental groups after thermocycling. An explanation might be that aging degradation induced cement debonding from microleakage. In particular, all specimens were debonded in the NT (+), C-NT (+), and C-ET (+) groups because etched and non-treated surfaces showed lower mechanical retention than sandblasted surfaces. Comparing effects of conditioner applications, the C-SB (+) group showed a lower decrease in SBS and a higher degree of mixed fracture compared with the SB (+) group. Thus, the higher mechanical retention of a sandblasted zirconia surface was effective for subsequent resin bonding as mentioned above. For the sandblasted zirconia surface, the combination of primers containing phosphate monomer and/or luting resins was recommended from clinical outcomes [14]. In an in vitro study, the application of various universal primers (adhesives) for chemical bonding was effective on sandblasted zirconia surfaces to improve bonding to resin cement after aging [13], as only sandblasted and silanated surfaces showed a significant decrease in the SBS values [32]. One universal primer product might affect hydrolytic degradation and thus nullify the advantage gained from increased micromechanical retention after aging. However, Monobond Plus (Ivoclar Vivadent GmbH, Ellwangen, Germany) did not influence the hydrolytic degradation [13]. For the sandblasted zirconia surface, the combination of Monobond Plus and MDP-based self-adhesive cement showed aging resistance [34,35]. Thus, the combination of Monobond Plus and Panavia SA Cement Plus Automix (Kuraray Europa GmbH, Hattersheim am Main, Germany) was used in this study.

To sum up, the C-SB group has the potential for both strong micromechanical interlocking and chemical bonding compared with all other experimental groups, and showed improvements in bond strength and failure mode.

Recently, different modification methods of pre-sintered zirconia for the improvement of bonding were reported in in vitro studies [9,36,37]. Sandblasting prior to zirconia sintering increased the surface roughness and bond strength to veneering ceramics compared with that after sintering [9]. Actually, there are risks of defects in the thin parts of pre-sintered zirconia substrates during sandblasting. Moreover, coating treatments using silica or zirconia powder on the pre-sintered zirconia did not cause a phase transformation by sintering, and enhanced the bond strength of veneering ceramics or cement [36,37]. Unfortunately, these coating approaches are comparably time consuming [14]. In contrast, the novel conditioner application to zirconia surfaces following sandblasting in our study was simple, safe, and economic compared with these methods, thus indicating an advantageous method. Together with the results of a previous study [19], the present results indicate that this approach may improve the bonding characteristics to both resin cement and veneering ceramics. Further studies are needed to clarify possible clinical applications of this conditioner.

## 5. Conclusions

Within the limitations of this in vitro study, the conclusions are as follows:

1. Conditioner application increased the surface roughness and improved the wettability.
2. Without thermocycling, sandblasted surfaces showed significantly higher bond strengths compared with non-treated and chemically etched surfaces, irrespective of conditioner application (*p* < 0.05).
3. Adhesive fractures were commonly observed in all experimental groups, except in the C-SB group.
4. Thermocycling favored debonding and affected the failure modes and bond strengths. Pre-failure was observed in all specimens of the NT (+), C-NT (+), and C-ET (+) groups during thermocycling.
5. However, the C-SB (+) group showed the highest bond strength (*p* < 0.05) and still the mixed fractures partially after thermocycling.

Thus, the combination of conditioner application before sintering and sandblasting after sintering showed the highest shear bond strength and indicated improvements concerning the failure mode.

## Figures and Tables

**Table 1:** Materials used.

Material	Product	Composition	Manufacture	Lot No.
Zirconia (Y-TZP)	Nacera Pearl 1	ZrO_2_ + HfO_2_ + Y_2_O_3_ (≥99.0%), Al_2_O_3_	Doceram	17337
Al_2_O_3_ particle	Klasse 30B/50 my	50 μm	Harnisch + Rieth	–
Acid etching	Porcelain Etch	Hydrofluoric acid (9.5%)	Ultradent	BBNGY
Universal primer	Monobond Plus	Ethanol, Methacrylted phosphoric acid ester	Ivoclar Vivadent	R03109
		3-trimethoxysilylpropyl, Methacrylate		
Cement	Panavia SA Cement	TMSPMA, Bis-GMA, TEGDMA, HEMA, MDP, NaF	Kuraray	BE0025
	Plus Automix (Universal, A2)	Silanated colloidal silica filler, Silanated barium	Europa	–
		glass filler, Peroxide, dl-Camphor-quinone		
		Hydrophobic aliphatic dimetacylate/aromatic		
		dimetacrylate, Catalysys		

TMSPMA, 3-trimethoxysilylpropyl methacrylate; Bis-GMA, bisphenol A-glycidyl methacrylate; TEGDMA, triethylene glycol dimethacrylate; HEMA, 2-Hydroxyethyl methacrylate; MDP, 10-methacryloyloxydecyl dihydrogen phosphate.

**Table 2:** Experimental groups.

Group (Code)	Conditioner Application	Surface Modification	Thermocycling (+)
C-NT	Yes	Non-treatment	No
C-ET	Yes	Etching	No
C-SB	Yes	Sandblasting	No
C-NT (+)	Yes	Non-treatment	Yes
C-ET (+)	Yes	Etching	Yes
C-SB (+)	Yes	Sandblasting	Yes
NT	No	Non-treatment	No
ET	No	Etching	No
SB	No	Sandblasting	No
NT (+)	No	Non-treatment	Yes
ET (+)	No	Etching	Yes
SB (+)	No	Sandblasting	Yes

**Table 3:** Quantitative analysis of the dried powder in weight percent (wt. %) by X-ray fluorescence (XRF) analysis.

Compund	SiO_2_	Al_2_O_3_	K_2_O	Na_2_O	RbO	Fe_2_O_3_	ZrO	PbO
wt. %	74.1	12.1	12.1	1.61	0.21	<0.07	<0.05	<0.02

**Table 4:** Surface roughness parameters of each experimental group (median).

Parameter	NT	C-NT	*p*-Value	ET	C-ET	*p*-Value	SB	C-SB	*p*-Value
*R_a_* (μm)	0.16	0.74	**	0.40	0.56	–	0.38	0.68	**
*R_max_* (μm)	2.00	8.95	**	4.61	7.88	*	3.36	6.96	**
*R_sk_*	0.56	1.80	**	−0.11	1.81	**	−0.51	0.22	**
*R_pm_*/*R_z_*	0.56	0.67	**	0.46	0.67	**	0.41	0.50	**

Asterisk marks indicate significant differences (** *p* < 0.01, * *p* < 0.05). *R_a_*, mean roughness; *R_max_*, maximum roughness depth; *R_sk_*, skewness; *R_pm_*/*R_z_*, the ratio of the average of the leveling depths of five consecutive lengths/the mean roughness depth. Abbreviations are shown in Table 2, respectively.

**Table 5:** Water contact angles of each experimental group (mean (standard deviation) in °).

Group	Non-Conditioner (°)	Conditioner (°)
NT	60.4 (8.6) ^a,A^	36.2 (3.8) ^b,B^
ET	54.9 (14.9) ^a,A^	39.8 (10.6) ^b,B^
SB	62.1 (7.8) ^a,A^	36.0 (14.1) ^b,B^

Results of statistical analysis are represented by upper and lower case letters. Different uppercase letters in the same row mean that the groups are significantly different (*p* < 0.05). Different lowercase letters in the same column mean that the groups are significantly different (*p* < 0.05). NT, non-treatment; ET, etching; SB, sandblasting.

**Table 6:** Number of pre-failures and shear bond strengths in experimental groups (median in MPa).

Group	Pre-Failure (*n*)	Shear Bond Strength (MPa)
NT	0	7.97 ^A^
ET	0	4.40 ^B^
SB	0	14.13 ^C^
C-NT	0	6.52 ^A^
C-ET	0	5.48 ^B^
C-SB	0	14.33 ^C^
NT (+)	5	0
ET (+)	1	2.14 ^a^
SB (+)	1	5.37 ^b^
C-NT (+)	5	0
C-ET (+)	5	0
C-SB (+)	1	9.12 ^c^

Results of statistical analysis are represented by upper and lower case letters. Different uppercase letters in the strengths without thermocycling are significantly different (*p* < 0.05). Different lowercase letters in the strengths with thermocycling are significantly different (*p* < 0.05). Abbreviations of each experimental group are shown in Table 2, respectively. (+) indicates thermocycled samples.

**Table 7:** Distribution (%) of failure modes in the experimental groups with and without thermocycling.

Group	Adhesive (%)	Mixed (%)	Cohesive (%)
NT	100	0	0
ET	100	0	0
SB	100	0	0
C-NT	100	0	0
C-ET	100	0	0
C-SB	60	40	0
NT (+)	–	–	–
ET (+)	100	0	0
SB (+)	100	0	0
C-NT (+)	–	–	–
C-ET (+)	–	–	–
C-SB (+)	75	25	0

Abbreviations of each experimental group are shown in Table 2, respectively. (+) indicates thermocycled samples.
